# Changes in D-dimer after initiation of antiretroviral therapy in adults living with HIV in Kenya

**DOI:** 10.1186/s12879-020-05213-1

**Published:** 2020-07-14

**Authors:** Chloe A. Teasdale, Cecilia Hernandez, Allison Zerbe, Duncan Chege, Mark Hawken, Wafaa M. El-Sadr

**Affiliations:** 1 Department of Epidemiology and Biostatistics, CUNY Graduate School of Public Health and Health Policy, 55 W125th Street, Room 543, New York, NY 10027 USA; 2 ICAP at Columbia University, Mailman School of Public Health, Columbia University, 22 W168th Street, New York, NY 10032 USA; 3 Department of Epidemiology, Mailman School of Public Health, Columbia University, 722 W168th Street, New York, NY 10032 USA

**Keywords:** Coagulation, D-dimer, ART initiation, Tuberculosis, Women

## Abstract

**Background:**

Increased coagulation biomarkers are associated with poor outcomes among people living with HIV (PLHIV). There are few data available from African cohorts demonstrating the effect of antiretroviral therapy (ART) on coagulation biomarkers.

**Methods:**

From March 2014 to October 2014, ART-naïve PLHIV initiating non-nucleoside reverse transcriptase inhibitor-based ART were recruited from seven clinics in western Kenya and followed for up to 12 months. Demographics, clinical history and blood specimens were collected. Logistic regression models adjusted for intrasite clustering examined associations between HIV viral load and D-Dimer at baseline. Mixed linear effects models were used to estimate mean change from baseline to 6 months overall, and by baseline viral load, sex and TB status at enrollment. Mean change in D-dimer at 6 months is reported on the log10 scale and as percentage change from baseline.

**Results:**

Among 611 PLHIV enrolled, 66% were female, median age was 34 years (interquartile range (IQR) 29–43 years), 31 (5%) participants had tuberculosis and median viral load was 113,500 copies/mL (IQR: 23,600-399,000). At baseline, 311 (50.9%) PLHIV had elevated D-dimer (> 500 ng/mL) and median D-dimer was 516.4 ng/mL (IQR: 302.7–926.6) (log baseline D-dimer: 2.7, IQR: 2.5–3.0). Higher baseline D-dimer was significantly associated with higher viral load (*p* < 0.0001), female sex (*p* = 0.02) and tuberculosis (*p* =  0.02). After 6 months on ART, 518 (84.8%) PLHIV had achieved viral load < 1000 copies/mL and median D-dimer was 390.0 (IQR: 236.6–656.9) (log D-dimer: 2.6, IQR: 2.4–2.8). Mean change in log D-dimer from baseline to 6 months was − 0.12 (95%CI −0.15, − 0.09) (*p* < 0.0001) indicating at 31.3% decline (95%CI −40.0, − 23.0) in D-dimer levels over the first 6 months on ART. D-dimer decline after ART initiation was significantly greater among PLHIV with tuberculosis at treatment initiation (− 172.1, 95%CI −259.0, − 106.3; *p* < 0.0001) and those with log viral load > 6.0 copies/mL (− 91.1, 95%CI −136.7, − 54.2; *p* < 0.01).

**Conclusions:**

In this large Kenyan cohort of PLHIV, women, those with tuberculosis and higher viral load had elevated baseline D-dimer. ART initiation and viral load suppression among ART-naïve PLHIV in Kenya were associated with significant decrease in D-dimer at 6 months in this large African cohort.

## Background

Increased levels of D-dimer, a marker of hyper coagulation, have been shown to be a strong predictor of morbidity and mortality among people living with HIV (PLHIV) both prior to and after initiation of antiretroviral therapy (ART) [1–3]. The Strategies for Management of Antiretroviral Therapy (SMART) study provided important insights into the correlation between coagulation and inflammatory markers in PLHIV and increased mortality. The study found that D-dimer and interleukin-6 (IL-6) were the strongest predictors of all-cause mortality among a population of relatively healthy PLHIV [4]. Other studies have also shown that elevated D-dimer levels in PLHIV both before and after ART initiation are correlated with greater risk of non-AIDS events and mortality in resource-rich [2, 5] and resource-limited settings among populations with advanced HIV disease [3, 6, 7]. PLHIV, including those on ART, appear to be at higher risk for venous thrombotic events [8, 9] and pro-coagulant states [10, 11] which have been linked to continued immunodeficiency and low level viral replication even after treatment initiation [4, 11].

While long-term ART, particularly with use of specific antiretroviral drugs, has been associated with some metabolic complications, including hyperlipidemia and myocardial infarction [12, 13], ART also has beneficial effects in decreasing inflammatory and coagulation biomarkers. Studies have shown declines in D-dimer levels following treatment initiation, and have further demonstrated that delayed ART, poor treatment adherence and interruption of ART may contribute to elevated coagulation markers and increased risk of death [4, 14–16]. Nonetheless, compared to HIV-negative persons, higher D-dimer levels have been shown to persist even in PLHIV who maintain viral suppression on long-term ART [14, 17]. Few studies have examined change in D-dimer resulting from ART initiation including examination of pre-treatment measures, and most research in this area has been conducted in resource-rich settings. In this analysis, we present an investigation of changes in D-dimer levels after ART initiation among a large cohort of adult PLHIV in Kenya.

## Methods

The Antiretroviral Therapy and Inflammatory and Coagulation Biomarkers (ARTIC) prospective cohort study was designed to examine the relationship between ART use and changes in biomarkers among adult ART-naïve PLHIV initiating treatment in Kenya (NCT02027480). Study participants included male and female PLHIV aged 18 years and older who were recruited from seven health facilities in Nyanza region of Kenya from March through October 2014. All participants were eligible for ART based on Kenyan national guidelines which changed over the course of the study from CD4+ cell count (CD4+) < 350 cells/mm^3^ or World Health Organization (WHO) stage 3 or 4 to CD4+ < 500 cells/mm^3^ regardless of WHO stage (as of July 2014) [18]. The recommended first line treatment regimen in Kenya at the time of the study was Tenofovir (TDF) + Lamivudine (3TC) + Efavirenz (EFV) [18]. Women who were currently pregnant were excluded. Kenyan national guidelines called for immediate initiation of tuberculosis (TB) treatment with ART initiation to begin after TB treatment was tolerated, at least within the first 2–8 weeks after start of TB treatment. The preferred ART regimen for those on TB treatment was TDF + 3TC + EFV [18]. Participants who were diagnosed with TB after the enrollment visit were analyzed according to their TB status at enrollment. The ARTIC study was approved by the Kenya Medical Research Institute (KEMRI) and the Columbia University Medical Center (CUMC) Institutional Review Boards (IRB).

Consented participants attended an enrollment visit followed by visits at two, six and 12 months. The enrollment visit included collection of demographic, medical history, physical examinations including height and weight measurements, and collection of blood and urine samples. Participants received routine care at participating health facilities per national guidelines including initiation of non-nucleoside reverse transcriptase inhibitor (NNRTI)-based ART regimens. At all study follow-up visits, clinical data were abstracted from medical charts including CD4+ cell count and ART regimens. At six and 12 months, physical examinations included measurement of weight and blood pressure, and collection of blood samples to assess biomarker and HIV viral load over time. Blood specimens were collected by venipuncture using vacutainer tubes. Within 12 h of collection, specimens were transported to the KEMRI/CDC laboratory where they were stored at − 80 C until thawed for batched biomarker evaluation at the University of Nairobi Institute of Tropical and Infectious Diseases Laboratory (Nairobi, Kenya). We report measures of HIV viral load copies/mL (Xpert® HIV-1 Viral Load, Cepheid AB, Solna, Sweden) and D-dimer in nanograms (ng)/mL (Nano-Check™ kits, Nano-Ditech, New Jersey, USA). The lower limits of detection was 40 copies/mL for viral load and 200 ng/mL for D-dimer.

In this analysis, we describe study population characteristics at enrollment by D-dimer level including sex, age, CD4+ cell count, viral load level, body mass index (BMI) and TB status. D-dimer was dichotomized as elevated (≥ 500 ng/mL) and normal/low (< 500 ng/mL) based on standard (non-age adjusted) cutoffs used to identify activated coagulation in patients [19]. BMI was defined as individual’s weight divided by the square of their height (kg/m^2^) and was categorized in accordance with the WHO guidelines [20]. TB diagnosis at the time of study enrollment was ascertained from clinical records. Chi-square tests for proportions and Wilcoxon tests for medians were used to assess the unadjusted association of D-dimer at baseline with participant demographic and clinical characteristics including sex, age, CD4+ cell count, viral load, BMI and TB at study enrollment. We also examined the correlation between age and baseline D-dimer as a continuous variable using Pearson correlation coefficients. We report unadjusted median D-dimer levels at baseline and 6 months after ART initiation overall and according to pre-treatment viral load, sex and TB status at enrollment. D-dimer was not normally distributed and was evaluated in models on the log10 scale. To estimate mean change in log D-dimer values from baseline to 6 months, analysis of variance (ANOVA) models (random effects linear models) adjusted for baseline D-dimer were used. We also estimated mean log D-Dimer change in participants according to baseline viral load, sex and TB status at enrollment; separate bivariable models (adjusted for baseline D-Dimer) were run to estimate mean D-Dimer change by levels of the baseline characteristic (not adjusted for other variables). Mean change estimates were back transformed to the original D-dimer scale and presented as percent change from baseline. For examination of TB at enrollment (identified through routine care), we conducted an additional analysis including participants diagnosed with TB between the baseline and 2 month study visit; however these analyses did not yield results differing from the analysis with only those diagnosed at the time of study enrollment and are not presented.

## Results

Among 685 ART-naïve adult PLHIV enrolled in the ARTIC study, 611 (89.2%) were included in this analysis. Forty-nine (7.2%) participants were lost to follow-up after enrollment and another 25 (3.6%) were missing D-dimer measures at either baseline or 6 months. As shown in Table 1, among the included participants, half (50.9%) had high D-dimer levels (≥ 500) at study enrollment and median D-dimer overall was 516.4 (interquartile range (IQR): 302.7–926.6). Most participants were female (65.5%) and median age was 34 years (IQR: 29–43). At baseline median CD4+ count was 326 cells/mm^3^ (IQR 194–442) and median viral load was 113,500 copies/mL (IQR 23,600-399,000); half (50.4%) of all participants had a viral load > 100,000 copies/mL. Among 31 (5.0%) participants with TB at study enrollment, 19 (61.3%) were male, 29 (93.5%) had pulmonary TB (16 of whom were smear positive and 13 were smear negative) and two (6.5%) had extra-pulmonary TB. In addition, five participants were diagnosed with TB between the enrollment and the six-month visit (four were pulmonary smear negative and one was extra-pulmonary); four between the enrollment and 2 month visit and one additional participant between the 2 and 6 month visits. Most participants (69.5%) had normal BMI (18.5–24.9). The proportion of women with high D-dimer levels at baseline was more than double that for men (*p* = 0.02). Elevated D-dimer levels were also associated with higher viral load, 56.6% of PLHIV with elevated D-dimer had baseline viral load > 100,000 copies/mL compared to 44.0% of those with normal/low D-dimer (*p* = 0.01). PLHIV with TB disease at baseline were also more likely to have elevated D-dimer (*p* = 0.02). BMI and CD4+ at study enrollment were not associated with elevated baseline D-dimer. Age was also not associated with elevated baseline D-dimer when examined as a dichotomous variable (*p* = 0.60) (Table 1), nor as a continuous variable (correlation coefficient (r) = 0.03, *p* = 0.52).

**Table 1:** Characteristics at study enrollment of ART-naïve treatment eligible adults (≥ 18 years) living with HIV in Kenya and starting ART in 2014 according to enrollment D-dimer level, normal/low vs. high (*N* = 611)

	Total		Normal/low D-dimer (< 500 ng/mL)		High D-dimer (≥ 500 ng/mL)		
	N	%	N	%	N	%	*p*-value
	611	100.0	300	49.1	311	50.9	
**Enrollment D-dimer**, median (IQR)	516.4 (302.7–926.6)		301.7 (199.0–397-6)		912.8 (690.2–1446.2)		< 0.0001
**Sex**							
Male	211	34.5	117	39.0	94	30.2	0.02
Female	400	65.5	183	61.0	217	69.8	
**Age (years),** median (IQR)	34 (29–43)		34 (29–43)		34 (29–43)		0.86
< 25	52	8.5	29	9.7	23	7.4	0.60
25–40	384	62.9	186	62.0	198	63.7	
> 40	175	28.6	85	28.3	90	28.9	
**CD4+ cells/mm3,** median (IQR)	326 (194–442)		335 (221–451)		319 (177–435)		0.17
> 500	78	12.8	39	13.0	39	12.5	0.42
350–500	186	30.4	98	32.7	88	28.3	
200–349	190	31.1	95	31.7	95	30.6	
< 200	156	25.5	68	22.7	88	28.3	
Missing	1	0.2	0	0.0	1	0.3	
**Viral load copies/mL,** median (IQR)	113,500 (23,600-399,000)		82,200 (15,800-294,000)		159,000 (33,400-501,000)		0.0001
< 1000 (<log 3.0)	30	4.9	18	6.0	12	3.9	0.01
1000–9999 (log 3.0–3.9)	57	9.3	34	11.3	23	7.4	
10,000–99,999 (log 4.0- < 4.9)	201	32.9	107	35.7	94	30.2	
100,000-1000,000 (log 5.0–5.9)	255	41.7	116	38.7	139	44.7	
> 1000,000 (>log 6.0)	53	8.7	16	5.3	37	11.9	
Missing	15	2.5	9	3.0	6	1.9	
**BMI,** median (IQR)	20.9 (18.9–22.9)		21.1 (19.1–22.9)		20.5 (18.6–23.0)		0.21
Underweight (< 18.5)	114	18.7	48	16.0	66	21.2	0.17
Normal (18.5–24.9)	425	69.6	219	73.0	206	66.2	
Overweight & Obese (≥25)	72	11.8	33	11.0	39	12.5	
**Current TB at study enrollment**	31	5.1	9	3.0	22	7.1	0.02

**Fig. 1:**
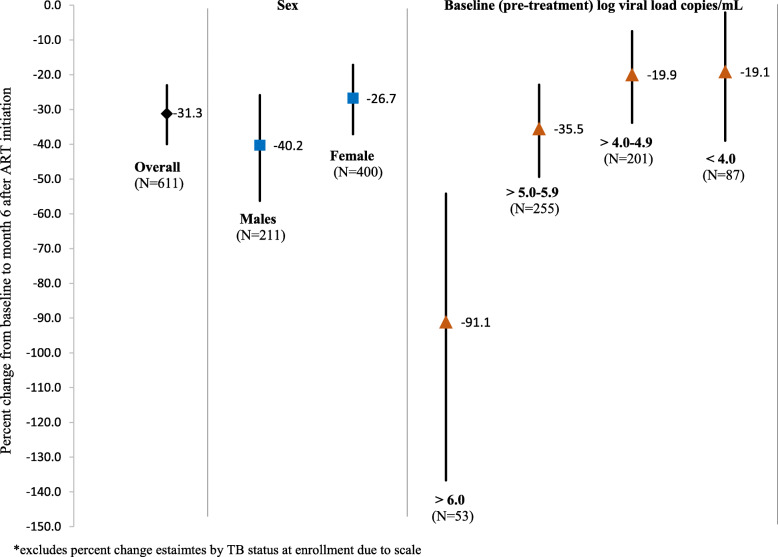
Mean percent change (95% CI) in D-dimer levels from baseline to 6 months after ART initiation among adults (≥ 18 years) starting ART in Kenya 2014 overall, and by sex and baseline log viral load (*N* = 611)*

At 6 months after starting ART, 518 (84.8%) PLHIV had achieved a viral load < 1000 copies/mL (5.7% missing viral load at 6 months). Median D-dimer at 6 months after ART initiation was 390.0 (IQR: 236.6–656.9) (log 2.6, IQR: 2.4–2.8) (Table 2). Mean log change for all participants from baseline to 6 months was − 0.12 (95%CI −0.15, − 0.09). Median D-dimer at baseline among PLHIV with log viral load > 6.0 /mL (819.8 ng/mL) and mean change at 6 months (− 0.28 (95%CI −0.37, − 0.19) was significantly higher compared to those with lower baseline viral load (*p* < 0.001) (Table 2). PLHIV with TB at enrollment had the highest baseline D-dimer (median 1230.3, IQR: 465.1–2586.3) and mean change in log D-dimer at 6 months in these participants was significantly higher than in PLHIV without TB (− 0.43, 95%CI −0.56, − 0.31, *p* < 0.0001). Although women had higher median D-dimer at baseline compared to men (566.1 vs. 448.0), there was no significant difference in mean change from baseline to 6 months in log D-dimer by sex (*p* = 0.14) (Table 2). Overall, there was a 31.3% decline in D-dimer from baseline to 6 months after ART initiation (95%CI −40.0, − 23.0) (Fig. 1). Among PLHIV starting ART with log viral load > 6.0 copies/mL, there was a 91.1% (95%CI –136.7, − 54.2) decrease at 6 months on ART, while those with baseline log viral load < 4.0, had a mean decrease of 19.1% (95%CI −39.0, − 2.1). PLHIV with TB at ART initiation saw the largest percent decline in D-dimer levels, 172.1% (95%CI −259.0, − 106.3).

**Table 2:** Median biomarker values at baseline and 6 months and mean change from baseline to 6 months among adults (≥ 18 years) starting ART in Kenya 2014 (*N* = 611)

	Baseline		6 months		Change baseline to 6 months	
	Median (IQR)	Log median (IQR)	Median (IQR)	Log median (IQR)	Mean log10 change (95% CI)	*p*-value*
**D-dimer** (ng/mL)	516.4 (302.7–926.6)	2.7 (2.5–3.0)	390.0 (236.6–656.9)	2.6 (2.4–2.8)	−0.12 (−0.15, −0.09)	< 0.0001
**Sex**						
Male	448.0 (245.7–872.1)	2.7 (2.4–2.9)	290.1 (199.0–539.3)	2.5 (2.3–2.7)	− 0.15 (− 0.2, − 0.1)	0.14
Female	566.1 (347.9–953.4)	2.8 (2.5–3.0)	430.2 (279.6–714.7)	2.6 (2.4–2.9)	− 0.10 (− 0.14, − 0.07)	
**Baseline log viral load** cells/mL						
> 6.0	819.8 (466.8–1262.3)	2.9 (2.7–3.1)	397.8 (259.5–617.8)	2.6 (2.4–2.8)	− 0.28 (− 0.37, − 0.19)	< 0.01
5.0–6.0	566.8 (320.2–970.4)	2.8 (2.5–3.0)	399.2 (246.1–723.3)	2.6 (2.4–2.9)	− 0.13 (− 0.17, − 0.09)	
4.0–4.9	461.8 (284.3–894.9)	2.7 (2.5–3.0)	410.3 (258.4–639.3)	2.6 (2.4–2.8)	− 0.08 (− 0.13, − 0.03)	
< 4.0	435.0 (258.7–683.1)	2.6 (2.4–2.8)	326.4 (199.0–590.6)	2.5 (2.3–2.8)	− 0.08 (− 0.14, 0.01)	
**Baseline TB**						
No	495.9 (300.6–884.8)	2.7 (2.5–2.9)	390.2 (237.4–666.3)	2.6 (2.4–2.8)	−0.10 (− 0.13, − 0.07)	< 0.0001
Yes	1230.3 (465.1–2586.3)	3.1 (2.7–3.4)	297.3 (199.0–572.8)	2.5 (2.3–2.8)	−0.43 (− 0.56, − 0.31)	

*p-values from Type 3 Tests of Fixed Effects

## Discussion

In this cohort of 611 ART-naïve, treatment-eligible Kenyan adults, almost half had elevated pre-ART D-dimer levels (≥ 500 ng/mL) indicating increased risk for morbidity and mortality. Women, PLHIV with higher viral load and those with TB at ART initiation were most likely to have elevated pre-treatment D-dimer. At 6 month visit after starting ART, D-dimer levels decreased, with the median falling to below the threshold indicative of elevated coagulation. The most significant declines were observed among those with highest pre-treatment levels, including participants with high pre-treatment viral load and those with co-existing TB. To our knowledge, this is largest cohort report of changes in this critical coagulation marker after ART initiation from a sub-Saharan African country and our findings provide important information about the benefits of treatment initiation, particularly for PLHIV with advanced disease.

At study enrollment, among all treatment eligible PLHIV in Kenya with median viral load of roughly 100,000 copies/mL, we observed a median D-dimer of 516 ng/mL which is above the clinical cutoff of 500 ng/mL. Our findings showed somewhat lower baseline levels than previous studies of untreated African cohorts, including a study of women living with HIV in Rwanda [21] and another of South African PLHIV with advanced disease [3]. In our cohort, women, PLHIV with high viral load and those with TB had higher pre-treatment D-dimer levels. Sex differences in coagulation makers have been previously documented in HIV-negative populations and in PLHIV enrolled in clinical trials conducted in high resource settings [22–24]. Similar to our results, a study conducted in South Africa and Uganda also found higher pre-treatment D-dimer levels in women compared to men [25]. Previous studies in PLHIV have shown an association between advanced disease and coagulation markers [1, 3, 22, 25], including D-dimer, however we believe this to be one of the largest African cohorts in which pre-treatment findings have been described. There are few previous reports of the relationship between TB and D-dimer in PLHIV however TB disease has been shown to induce a hypercoagulable state [26, 27] and a study of 98 PLHIV in Gabon also reported higher pre-ART D-dimer levels among those with concurrent TB infection compared to those without [28].

We report significant reduction in D-dimer levels over the first 6 month after ART initiation in Kenyan PLHIV, particularly among those with higher viral load and TB at enrollment. Overall, median D-dimer level at 6 months on treatment was 390 ng/mL which is below the clinical cutoff of 500 ng/mL and there was a 31% decline overall in D-dimer levels after treatment start. PLHIV starting ART with higher viral load and those with TB saw the largest declines in D-dimer. Our results are unique as few studies have evaluated change in coagulation markers after ART initiation among PLHIV coinfected with TB. While we had only a small number of participants with TB at enrollment (*N* = 31), in this cohort, PLHIV with TB had significantly higher pre-ART D-dimer levels compared to those without TB and experienced a 172% decline in D-dimer, with median D-dimer levels at 6 months within clinically normal values. Our findings are consistent with the previously cited study from Gabon which also found significant decreases in D-dimer among 19 PLHIV with TB after ART initiation [28]. These findings demonstrate the important benefits of treatment initiation for PLHIV particularly those with advanced disease and comorbidities.

While the impact of treatment with antiretroviral medications on levels of coagulation markers in PLHIV has been shown in clinical trials, there are limited findings from real world cohorts, particularly from African settings. The SMART trial found that among participants who achieved viral load < 400 copies/mL, at 6 months on ART median decline in D-dimer level was − 0.10 μg/mL (IQR: − 0.31 – 0.00) or a 51% reduction [15]. The Monitoring of Early Adherence (META) cohort study, conducted in South Africa and Uganda, also showed significant reductions in D-dimer at 12 months after ART initiation among 438 PLHIV [25]. While we examined D-dimer change over the first 6 months on ART in a cohort of PLHIV with more advanced disease than these previous studies, our results are consistent. Our estimate of mean change in the first 6 months after treatment initiation is also similar to a study of 100 PLHIV from South Africa with advanced disease which reported significant reductions in D-dimer at 6 months after ART initiation (− 0.12, *p* < 0.0001) [3]. In a cohort of Rwandan women living with HIV, significant reductions in D-dimer levels were observed at two years on ART demonstrating the continued salutary effect of treatment initiation [21]. The decrease in D-dimer levels as a result of treatment can be anticipated to result in favorable outcomes in terms of thrombotic complications and survival.

There are several important strengths of this analysis. As noted, our findings are unique based on the size and setting of the cohort, as well as the measurement of D-dimer and viral load over the first 6 months of treatment. The large size of this African cohort is important given that the majority of PLHIV receive care in similar settings and the fact that few prior studies have focused on coagulation markers in this population. Our findings demonstrating the effect of ART initiation on D-dimer levels are highly relevant for this population and underscore the importance of treatment initiation to improve long term health outcomes for PLHIV. Our study is also novel with regard to our description of D-Dimer in PLIHV with TB disease; as noted, very few previous studies have examined D-dimer in PLHIV with TB despite it being the leading cause of death among PLHIV [29]. There are also limitations to our analysis including the relatively short duration of follow-up which did not allow us to examine the durability of the reductions we observed in D-dimer. The sample size also limited our ability to examine associations between baseline and change in D-dimer levels with morbidity and mortality outcomes over time. We did not have a comparison group with which to compare our data such as PLHIV starting ART with higher CD4 counts or persons not living with HIV. Our study was conducted prior to the introduction of ‘treat all’ guidelines in Kenya and during a time when majority of patients initiated NNRTI-based regimens. This cohort is thus representative of the patient population eligible for treatment during that period and reflect outcomes for PLHIV starting the ART regimens recommended at that time.

## Conclusion

In summary, while we found important associations with D-dimer levels and baseline characteristics as well as significant reductions in D-dimer following ART initiation, it should be noted that women and those with higher pre-treatment viral load appear to have persistently elevated levels which suggests continued risk for comorbidities including cardiovascular disease (CVD), and may be indicative of ongoing mortality risk [26]. Further research is needed to identify interventions that are effective at decreasing non-AIDS related morbidity and mortality among PLHIV on ART.

## Data Availability

Data from the ARTIC study are available upon request; please contact Allison Zerbe at ICAP at Columbia University, az2258@columbia.edu

## Abbreviations

3TC: Lamivudine
ART: Antiretroviral therapy
ARTIC: Antiretroviral Therapy and Inflammatory and Coagulation Biomarkers
BMI: Body mass index
CDC: US Centers for Disease Control and Prevention
CUMC: Columbia University Medical Center
EFV: Efavirenz
HIV: Human immunodeficiency syndrome
IQR: Interquartile range
IL-6: Interleukin 6
IRB: Institutional review board
KEMRI: Kenya Medical Research Institute
NNRTI: Non-nucleoside reverse transcriptase inhibitor
mL: Milliliter
ng: Nanograms
NIH: National Institutes of Health
OAR: Office of AIDS Research
PLHIV: People living with HIV
TB: Tuberculosis
TDF: Tenofovir
VL: Viral load
WHO: World Health Organization
